# The Water Supply of Buffalo

**Published:** 1912-05

**Authors:** 


### BUFFALO MEDICAL JOURNAL

**A Monthly Review of Medicine and Surgery**

*EDITOR*

A. L. BENEDICT.

All communications, whether of a literary or business nature, books for review and exchanges, should be addressed to the editor, 354 Franklin Street, Buffalo, N. Y Please make personal or telephonic calls before 1 p. m

The Buffalo Medical Journal will publish regularly. full transactions of any professional organization in western New York at cost. Personal notices, brief reports of society proceedings, and the like, are always welcome and will be published withou charge.

Subscriptions may commence at any time. $2.00 per year in advance,

Vol. LXVII. MAY, 1912 No. 10

*Sanitary Science is mainly applied decency.*

### The Water Supply of Buffalo

One of Col. Potter's editorials, reported in abstract frem the issue of February, 1907

The question of pure water supply for Buffalo is again being agitated. The people of the city, if they have anything to say about the matter at all, say it in the privacy of their own homes when they see the muddy water which is their portion after every little wind storm, and they say what they have to say where it can not affect their standing as christians and law-abiding people. It is difficult to understand just why Buffalo, situated as it is, at the very threshold of unlimited supply, should be so ill-provided with so necessary an article as pure water.

There has been water talk enough to saturate a dozen cities of the size of Buffalo.

The latest effort to get something like a human idea of what the situation really is—the consideration of the water question in its broadest sense by the Academy of Medicine at a recent meeting,—was productive of nothing at all except a triple row of damnations from Col. Ward, the Commissioner of Public Works.

St. Louis, Boston, Washington and other cities have been through the same trouble; the only difference is that these cities have met the difficulty promptly—whether adequately or not, need not be here considered at this time.

What Buffalo needs is civic surgery.

At present there is every probability that the proposed new intake to the Emerald channel will be three years building. 'Till then, Buffalo will suffer for water.

